# Association between Work-Related Stress and QT Prolongation in Male Workers

**DOI:** 10.3390/ijerph16234781

**Published:** 2019-11-28

**Authors:** Luigi Isaia Lecca, Igor Portoghese, Nicola Mucci, Maura Galletta, Federico Meloni, Ilaria Pilia, Gabriele Marcias, Daniele Fabbri, Jacopo Fostinelli, Roberto G. Lucchini, Pierluigi Cocco, Marcello Campagna

**Affiliations:** 1 Department of Experimental and Clinical Medicine, University of Florence Largo Piero Palagi 1, 50139 Florence, Italy; nicola.mucci@unifi.it; 2 Department of Medical Sciences and Public Health, University of Cagliari, Blocco I, SS 554, km 4,500, 09042 Monserrato, Italy; igor.portoghese@gmail.com (I.P.); maura.galletta@gmail.com (M.G.); federicomeloni@hotmail.it (F.M.); cromatinap@gmail.com (I.P.); gabriele.marcias@libero.it (G.M.); daniele.fabbri@hotmail.it (D.F.); pcocco@unica.it (P.C.); mam.campagna@gmail.com (M.C.); 3 Department of Civil and Environmental Engineering and Architecture, University of Cagliari, 09123 Cagliari, Italy; 4 Occupational Medicine, University of Brescia, P.le Spedali Civili 1, 25123 Brescia, Italy; jacopo.fostinelli@unibs.it (J.F.); roberto.lucchini@unibs.it (R.G.L.); 5 Division of Occupational and Preventive Medicine, Icahn School of Medicine at Mount Sinai, New York, NY 10029, USA

**Keywords:** work-related stress, autonomic nervous system, QTc interval, QT index, cardiovascular diseases

## Abstract

Background: Work-related stress is a potential cardiovascular risk factor, but the underlying mechanism is not fully explained. The autonomic nervous system control of cardiac function might play a specific role; therefore, monitoring the QT interval in the electrocardiogram can highlight an autonomic imbalance induced by occupational stressors. The aim of our study was to explore the QT interval parameters as early indicators of imbalance of the autonomic cardiac function in relation to work-related stress. Methods: During 2015–2016 annual workplace health surveillance, we measured work-related stress in 484 workers of a logistic support company using the Health and Safety Executive (HSE) tool. We assessed the frequency-corrected QT (QTc) interval and the QT index (QTi) on the electrocardiogram of each participant, and collected demographic and clinical data. We compared the QTc values by the four Karasek’s categories (active/passive jobs, low/high strain job), and by job support (present/lacking), and conducted multivariate analysis to adjust for possible confounders. Results: The results of the multivariate regression analysis showed that QTc was prolonged among workers operating at a specific site where stress level was found to be elevated. Regular physical activity showed a beneficial effect against QTc prolongation. We did not observe an effect on QTc length by the cross-combined Karasek’s categories of job control, job demand, and job support. Conclusions: Our study suggests subclinical effects of conditions associated with work-related stress on the autonomic regulation of cardiac function. Further research is warranted to elucidate the combined effect of work organization and lifestyle factors on autonomic cardiac function.

## 1. Introduction

### 1.1. Linkage between Stress and Cardiovascular Disease

Work-related stress is a potential risk factor for the development of cardiovascular disease (CVD) [1,2,3], a leading cause of morbidity and mortality, and therefore a serious social problem, and an economic burden for health care systems [4,5,6]. The economic impact of work-related stress is also relevant in itself, because of the elevated number of sick leave days, the related decrease in work productivity, and the resulting loss for the economy [4].

The mechanism linking stress to CVD development is not fully understood; complex connections between the autonomic nervous system and the hypothalamic–pituitary–adrenal axis may be involved [1,6,7], with an immediate effect on heart rate by the sympathetic and parasympathetic nerves’ intrinsic activity on the sinoatrial node. The first results in increasing, and the second in decreasing heart rate. Secondly, the activation of the sympathetic nervous system promotes the release of catecholamines through the hypothalamic–pituitary–adrenal axis, which prolongs the effect on heart rate and heart rate variability (HRV) elicited though the neural route response [7]. On the other hand, exposure to the major CVD risk factors is associated with a decrease in vagal function, suggestive of disruption of the autonomic nervous system [8]. Whether the QT interval parameters calculated on the electrocardiogram tracing (ECG) predict risk of sudden death is controversial [9,10]; still, their use has been proposed along with heart rate variability (HRV) as biomarkers of stress-related changes in the early detection of autonomic imbalance [11,12,13]. However, while HRV has not always been recognized as a CVD predictor [14,15], the heart rate-corrected QT parameters (QTc = QT corrected, and QTi = QT index) have been used as biomarkers of autonomic dysfunction following work-related stress. For instance, Collins et al. (2010) found an association between high strain job and changes in the cardiac vagal function [16], and Maeda et al. (2015) found a positive link between QT index and domestic, but not work-related stress [17]. Measurement of the ECG QTc interval was originally proposed to predict the polymorphic ventricular tachycardia, known as Torsades de pointes, consisting of oscillations of the QRS complex around the isoelectric line. This syndrome is associated with the occurrence of syncope episodes, with possible evolution to ventricular fibrillation and sudden death [18]. Various factors can modify the QT interval, including intake of drugs that can interfere with ion flow through the K^+^ channels, a risk factor for syncope episodes and sudden death [18]. The hereditary long QT syndrome (LQTS) seems to be associated with polymorphisms in the KCNQ1 gene, located in the short arm of chromosome 11 (LQT1), and in the KCNH2 and SCN5A genes located in chromosomes 3 and 7, and associated with LQT2 and LQT3, respectively. These three gene polymorphisms account for about 75% cases of hereditary LQTS [19]. Workplace factors, such as shiftwork in irregular and unpredictable working hours, can induce changes in the length of the QT interval [20]. QTc changes have also been associated with specific metabolic profiles in a cohort of shift workers engaged in nonstandard working hours [21].

### 1.2. Theoretical Background: Organizational Factors and Stress

Flight communication and safety includes a complex array of different job tasks in a high security area, which require high coordination and high responsibility. The scrupulous respect of rules, procedures, and guidelines limits the personal control over job operations; such organizational factors might induce work-related stress [22]. Besides, as required by the specific workplace, the same job can imply a regular, constant activity or alternating periods of high and low flight traffic. This second scenario, typical of companies providing their service in tight schedules followed by low activity periods, would imply a periodically excessive workload to the personnel involved, matching high job demand with low job control, a potentially stressful condition [22]. Assessment of work-related stress takes advantage of validated, self-administered questionnaires, such as those reliably used for the Effort/Reward Imbalance (ERI) [23], and the Job Demand and Control (JDC) models [24]. Several theoretical models have served as a frame for research on job dissatisfaction, burnout, turnover, and work-related physical and psychological outcomes, such as CVD, sleep disorders, depression, and anxiety [12,13,16,17,25]. 

Karasek identified job demand and job control as two key factors affecting well-being in the workplace [24,26,27]. Work-related stress results from the combination of these two dimensions, particularly in a highly demanding job with poor job control. In the Job Demand and Control (JDC) model, the first is defined in terms of workload and time pressure; job control, also termed decision latitude, is defined as the workers’ ability to control their tasks and the general work process. Karasek suggested combining the two dimensions to classify jobs in four different categories: high strain (jobs high on demands and low on control), low strain (jobs low on demands and high on control), passive (jobs low on demands and low on control), and active jobs (jobs high on demands and high on control) [26]. Karasek subsequently extended the JDC model and its taxonomy by integrating job support as a third component [27]: highly demanding jobs with low control and low social support would be mostly harmful for workers’ well-being [28].

### 1.3. Objectives

Since flight logistic workers are potentially exposed to work-related stress and to organizational factors with a putative role on the onset of stress and related adverse health outcomes, there is a need for studies that consider these aspects in a unique framework. To the best of our knowledge, thus far, no studies have considered the influence of different job modalities on stress and related adverse health outcomes, and particularly on autonomic cardiac function. 

We investigated whether early indicators of autonomic cardiac function imbalance, as detected by routine ECG, would vary by Karasek’s taxonomy categories, and other workplace and individual conditions. We focused on changes in QT interval parameters among workers free of overt cardiovascular dysfunction, also considering potential confounders.

## 2. Materials and Methods 

### 2.1. Study Population

During the 2015–2016 workplace health surveillance program, we recruited 486 white Caucasian male workers engaged in testing communication and flight safety systems in two locations within the region of Sardinia (Italy). The selected population represents about 50% of the company workforce, accounting for over 1000 workers equally distributed in the two locations. Therefore, the study population can be considered as a representative sample of the company workforce, providing enough reliability to the results. Other cross-sectional studies of similar size investigated the relationship between stress and related adverse health outcomes in other jobs [29]. Subjects from location 1 (*N* = 280), who operate by scheduled test campaigns with intervals of variable duration, were recruited in January–February 2015; subjects from location 2 (*N* = 206), who operate with daily operational activity, were recruited in November–December 2016. Shiftwork schedules were the following: fixed daytime shifts (daytime, including administrative and support work tasks and flight control personnel); 12 h shifts (H12, technical support personnel, such as electricians, radar operators and firefighters with a DNRRDN rotating shift schedule); and a 24 hour shift followed by a 96 hour rest (H24, security personnel).

Inclusion criteria were the following: being at work for one year or longer at the time of the 2015–2016 workplace health surveillance; availability of the ECG recorded during the last annual workplace health surveillance; and male gender. Females were few (<5%), and concentrated mainly in technical support tasks, with only one in security jobs involving night shifts. For these reasons, and because of gender differences in QT-related parameters and reaction to stress [30,31], we excluded them from the study. Other exclusion criteria included the following: prior diagnosis of any cardiovascular disease (ischemic heart disease, arrhythmia, cardiac hypertrophy, hypertension), diabetes, any neoplastic disease, and being under medication with drugs potentially causing a prolonged QT interval, as reported by the updated “QTDrugs Lists” of the Internet platform CredibleMeds [32]. Overall, we excluded 39 units of administrative staff employed in a fixed daytime shift (65% of the total excluded subjects), 14 subjects in H12 shift work (23%), and seven in H24 shift work (12%). The acceptance rate was very high (99.8%), as only one subject refused to fill the questionnaire. Therefore, we conducted the analysis in 425 male workers.

We conducted the study in accordance with the Helsinki Declaration. The study protocol included a self-administered questionnaire, a clinical exam, a routine electrocardiogram (ECG), and routine blood and urine tests, as approved by the Ethics Committee of the Cagliari University Hospital (Prot. PG/2017/ 16726). Prior to undergoing the study protocol, we informed all the eligible participants about the purpose of the study, and those who accepted participation signed an informed consent form. Questionnaires were anonymous and were coded and stored separately from the clinical records of each participant. The PI (MC) was responsible for safe storage of the file linking individual data to the i.d. codes. 

### 2.2. Demographics and Clinical Records 

Data abstracted from the medical chart included age, duration of employment, job tasks, type of shift schedule, height, weight, heart rate at rest, and systolic and diastolic blood pressure. A copy of the ECG tracing was also available for all the study subjects. 

The thresholds to define bradycardia and tachycardia were 50 and 100 beats per minute (bpm), respectively. Systolic (SBP) and diastolic blood pressure (DBP) were registered by a trained medical doctor during the clinical exam, using a manual sphygmomanometer, after holding the supine position for 2 minutes. Hypertension was defined as SBP ≥ 140 mmHg, or DBP ≥ 90 mmHg. We used the WHO criteria to define overweight (BMI > 25 kg/m^2^) and obese subjects (BMI > 30 kg/m^2^). Data on lifestyle habits (smoking, alcohol intake, coffee intake, and recreational physical activity) were part of the self-administered questionnaire. Recreational physical activity was defined as regular if the subject exercised at least twice a week.

### 2.3. Assessment of QT Interval Variables

We used a portable electrocardiograph (P80 Power-ESAOTE, Florence, Italy) to perform ECGs between 07:00 a.m. and noon, after resting in the supine position for 3 minutes in an ambulatory room with a temperature of 24 °C and no direct light illumination (screened windows) to allow acclimatization. Each subject had been off work or working only during the daytime the previous day, to ensure a restful condition during the test. No other source of stress nor environmental exposures, such as noise or airborne emissions in the workplace, were present. ECG recording lasted about two minutes; when reaching the perfect stability of the signal at visual monitoring, a trace without artifact was printed and stored. The equipment automatically calculated QTc by dividing the QT interval by the square root of the RR interval in milliseconds (ms), according to the Bazett’s formula (QTc = QT/√RR) [33], which represents the most used method to correct QT duration by heart rate, and has been used in several experimental and clinical studies [17,30]. A trained medical doctor (LIL) visually validated the measure, as the distance between the onset of the QRS complex and the end of the T wave in the precordial V1–V6 derivations, as recommended by the American Heart Association Electrocardiography and Arrhythmias Committee, the Council on Clinical Cardiology, the American College of Cardiology Foundation, and the Heart Rhythm Society [34]. Consistent with the reference values reported in the literature, a QTc shorter than 430 ms was considered normal; it was borderline between 430 and 450 ms; and prolonged if longer than 450 ms, [18]. Since QTc value might be affected by underestimation of QT duration, particularly at low frequency rate, QTi was also measured, which offers a reliable correction also for the for the heart frequency rates at which QTc is less reliable [35]. Therefore, the same examiner manually calculated QTi using the Rautaharju formula [35], as the percent ratio between measured and predicted QT (pQT). The predicted QT value was calculated as it follows [31]:

pQT = 656/(1 + 0.01 heart rate)

QTi values were considered abnormal when ≥105, and borderline when between 100–104 [17]. 

### 2.4. Assessment of Work-Related Stress 

The self-administered questionnaire included the Health and Safety Executive (HSE) indicator tool, and the subscales of job demand (eight items), job control (six items) and support from coworkers (horizontal support; four items) [36] to assess work-related stress. To test internal reliability, we calculated the Cronbach alpha, a measure of internal consistency that refers to the degree of interrelatedness among the items [37]. The values were 0.84, 0.81, and 0.83 for the three subscales, respectively, indicating that the items have a high internal consistency. 

Using the median scores for job demand and job control from the HSE questionnaire as cutoffs, we grouped study subjects into the four Karasek’s job taxonomy cells, of high/low job strain, and active/passive job, and by job support from peers [27]. 

### 2.5. Statistical Analysis 

We used parametric and nonparametric statistics, as appropriate, to test the differences across the four Karasek’ categories, by job support and operating site. A fixed daytime work schedule was the reference category for shift work; the low category was the reference for job demand; the high category was the reference for job control, and job support; abstinence was the reference for alcohol intake, current nonsmoking (including former smokers) for smoking, and physical inactivity for recreational physical activity. We assessed the correlation between parametric and nonparametric variables with the Pearson’s or Spearman’s correlation coefficient, as appropriate. We also conducted multiple regression analysis to predict QTc values as a function of the variables that decreased the residual variance of the model. These were: work-related stress dimensions (job demand, job control and job support scores), age, education, operating location, duration of employment, shift work schedule, smoking habit, alcohol intake, body mass index (BMI), SBP, DBP, and recreational physical activity. The multiple regression model predicting QT parameters and their 95% CI in the eight subcategories of the four Karasek’s taxonomy categories stratified by the two job support categories, included the following covariates: operating location, physical activity, SBP, age, education, and alcohol intake. Finally, we tested the observed differences across the estimated QT values by the analysis of variance (ANOVA). We rejected the null hypothesis when the α error probability associated with the statistics was lower than 5%. The analysis was conducted with SPSS^®^ v20.0 (SPSS Inc., Chicago, IL, USA).

## 3. Results

Table 1 shows selected characteristics of the study population by operating site. Study subjects in the two sites differed substantially by age, duration of employment, QTc, and QTi. The mean BMI (25.79 kg/m^2^) exceeded the threshold value for overweight; obese subjects (BMI > 30 kg/m^2^) accounted for 6% (*n* = 25) of the participants. The prevalence of smokers in the overall population was 27.1% (26.4% in site 1 vs. 27.9% in site 2). The median score of the work-related stress dimensions was 14 (range 7–37) for high job demand, 22 (range 9–30) for low job control, and 16 (range 3–20) for low job support. Subjects from the two sites had similar scores for the three work-related stress dimensions (Kruskal–Wallis test: *p* = 0.187; *p* = 0.767; *p* = 0.089 for job demand, job control, and job support, respectively).

The average QTc and QTi values were slightly elevated among subjects excluded with reference to those included in the study (mean QTc: 410.94 msec. *sd* 27.31 vs. 401.37 *sd* 25.91, *p* = 0.009; QTi: 99.25 *sd* 6.01 vs. 97.37 *sd* 5.61; *p* = 0.017). Compared with the reference values, about 10% (*n* = 43) and 3% (*n* = 11) of the study subjects had a borderline or abnormal QTc, respectively, and 9% (*n* = 37) had an abnormal QTi value (not shown in the tables).

Table 2 shows the correlation matrix of parametric and nonparametric variables, respectively. QT related parameters tended to increase with age (QTc: *p* = 0.018; QTi: *p* = 0.013), but not with BMI. QTc, but not QTi, showed a positive correlation with SBP (*p* = 0.026) and DBP (*p* = 0.037), and it was also significantly longer among participants in location 2 (*p* = 0.002), and inversely correlated with education (*p* = 0.016), and recreational physical activity (*p* = 0.006). 

HR showed a positive correlation with BMI (*p* = 0.003), SBP (*p* = 0.002) and DBP (*p* = 0.004). In turn, BMI was strongly correlated with SBP (*p* = 0.8 × 10^−5^) and DBP (*p* = 1.7 × 10^−9^), as seen in Table 2.

As expected, a highly demanding job was inversely correlated with job control (*p* = 3.52 × 10^−7^), and job support (*p* = 1.9 × 10^−7^). The job control score was significantly lower among study subjects engaged in H24 shift work (*p* = 3.9 × 10^−5^), and in regular recreational physical activity (*p* = 0.046), and it was directly correlated with age (*p* = 0.0001) and duration of employment (*p* = 0.031). On the other hand, physical activity was inversely correlated with BMI, DBP, heart rate at rest, and smoking (*p* = 0.027, 0.010, 1 × 10^5^, and 4 × 10^−4^, respectively).

The proportion of subjects with borderline and abnormal QTi and QTc values did not vary by categories of job demand, job control, job support, smoking habit, shift work, and operating location (not shown in the tables). 

The multiple linear regression model predicting QTc indicated that working in site 2 (β = 6855; *p* = 0.008) was the major contributor to a prolonged QTc interval, whilst physical activity (β = −6947; *p* = 0.007) tended to reduce it, as seen in Table 3. The analysis by operating site confirmed the protective role of physical activity on QTc length (β = −6647; *p* = 0.048 for site 1, and β = −8143; *p* = 0.047 for site 2), and education (β = −8216 *p* = 0.043) for site 2, as seen in Table 4. 

Table 4 shows the values of QTc predicted by the multivariate regression models, in each cell of Karasek’s category and job support, corrected by operating site, mean age and SBP, and median education level, alcohol intake, and recreational physical activity. and. The analysis of variance did not show significant differences in the predicted QTc value by Karasek category and job support category, and among the eight subgroups altogether.

## 4. Discussion

A prolonged QTc and QTi conveys an increased risk of primary cardiac arrest among persons without clinically recognized heart disease [38,39]. As work-related stress has been linked to CVD risk [1,2,3], monitoring QT-related parameters (QTc, QTi, QT variability, QT dispersion) might be useful to prevent a major cause of death, disability, and loss of productivity. By reaching a large fraction of the general population, the periodical occupational health surveillance [17,20,21,30,40] is a point of intervention: in fact, it might allow more effective preventive action through detecting early changes in biological parameters in otherwise healthy subjects. This aspect might have important public health implications, by reducing the direct and indirect costs of cardiovascular disease related to work-related stress. 

Our findings confirm the hypothesis that multifactorial external and internal events are capable of interfering with the cardiac autonomic balance. Variations in the QT interval parameters seem to reflect these effects. Concerning work-related stress dimensions (demand, control, and support), the median scores in our logistic support workers are alike to those reported in another study of a mixed population that used the same HSE indicator tool [41]. After multiple adjustments, we were unable to detect significant changes in the QTc interval across the combination of the four Karasek’s dimensions with the two categories of job support. 

Despite belonging to the same company, several differences were identified between the study population at the two work sites, in terms of mean age, shift work engagement, and duration of employment in the specific site, as well as work organization (scheduled high activity periods followed by low activity in site 1, vs. regular work activity in site 2). It is noteworthy that site 2 workers, who were more frequently engaged in h12 shifts, including night shifts, had a significantly prolonged QTc interval compared to workers in site 1. This finding would support those from previous reports showing that the type of work shift schedule affects QTc interval [20]. The work organization in site 2, that operates in a continuous way, may also contribute, and not by campaigns as in location 1. However, this would be counterintuitive, as a sense of underemployment and low job satisfaction might arise in workers not regularly employed in operative tasks [42,43]. Besides, job support was apparently lower in site 2, where longer mean QT parameters were detected. This borderline significant finding, although not confirmed by the multivariate analysis, might be cautiously explained by a possible mediating role of job support in the relationship between stress and health outcomes [44,45], when it acts in combination with other influencing factors, either organizational or personal. 

Age was a clear contributing factor in QTc and QTi prolongation in our study. Consistent with other studies, this finding supports the influence of aging on cardiovascular parameters [20,46]. This aspect is of special interest considering the constant, progressive aging of the working population in various lines of work [47], which elevates the importance of assessing inexpensive, and simple-to-apply early biomarkers, such as the QT parameters, to predict adverse CV outcomes.

The role of regular recreational physical activity in preventing QT prolongation was previously reported [48]. Consistent with other studies [30,40], such an effect was less evident for QTi, which would suggest that the two indexes might reflect the effect of different conditions. Besides, QTi might be an indicator of autonomic dysfunction caused by work stress more useful than QTc, being less sensitive to heart rate and HRV parameters [30]. Therefore, we propose using both in order to attain a greater sensitivity.

We could not detect any clear health effect related to the work conditions in our study. In fact, we aimed to investigate early alterations of the cardiac autonomic control, before the onset of overt CVD, with a prevention perspective. Therefore, we excluded all the subjects with an overt CVD. Based on our findings, we are planning further investigation with a prospective study design to explore whether organizational factors would interact with personal conditions in increasing the odds for specific diseases, including CVD.

Some aspects in the study design and peculiarities in the study population can limit the interpretation of our findings. First, we conducted a cross-sectional study, which does not allow a precise quantification of risk of developing changes in QT parameters attributable to work-related stress. The turnover was particularly rapid among workers in location 2, whose average duration of employment was around 3 years. This might be an indicator of stressful conditions, which might contribute to the longer QTc interval observed in this subgroup, and it might have prevented our ability of detecting effects on the stress dimensions, because of the relocation of workers who manifested stress symptoms. This is a typical drawback of the cross-sectional study design, such as the one we adopted in our study. In particular, an underestimation of the association between occupational stress and QT parameters can result if subjects with a risk profile, including those with a prolonged QT interval, have been moved to a less stressful environment. A prospective study design with multiple assessments of stress and QT parameters would be the proper strategy to prevent such bias from occurring. Despite this, we tentatively suggest that a combination of organizational factors (workload scheduling, shiftwork modality, job support), personal factors (age, physical activity, educational level), and other factors we could not assess in our study might contribute to modifying specific cardiac outcomes [49] 

Secondly, we had to exclude the female personnel because their small number would not have allowed any profitable gender-specific analysis. Therefore, it is uncertain whether our findings would also apply to the female population. Moreover, we did not consider the contribution of domestic stress, mainly related to family issues such as providing care for the elderly, disabled relatives, or children. Marital status, number of economically dependent family members, financial issues, and sleep disorders might also play a role in generating stress, particularly when interacting with adverse working conditions. If such aspects substantially differed between the two subgroups of our study population, bias might have affected our findings. 

## 5. Conclusions

In spite of some limitations, our results suggest a plausible link between aspects related to stress at work and changes in markers of early autonomic dysfunction of the cardiovascular system. Also, our observations are consistent with, and add to previous reports showing the contribution of work-related stress to the pathophysiological deregulation of the autonomic nervous system, leading to an increase in CVD risk [16,29,50,51]. We observed a significant QT prolongation among workers in one of the two operating sites in our study, which operated under a different work organization scheme. In conclusion, our findings suggest that organizational factors, along with personal lifestyle factors and occupational stress, may contribute to early alterations of autonomic cardiac control, such as a prolonged QT interval. Organizational factors such as workload schedules, shift work, and job support from peers can represent targets of intervention to manage work-related stress, and, consequently, the general health conditions among workers. 

Further studies with a larger sample size might determine the combined effect of work organization and lifestyle factors on the autonomic nervous system and the cardiovascular system.

## Figures and Tables

**Table 1:** Selected characteristics of the study population (overall study population and by company location).

Continuous Variables, Mean (sd)	Overall Study Population *N* = 425	Site 1 *n*. 246	Site 2 *n*. 179	*p*-Value
Age (years)	42.4 (7.58)	40.9 (8.14)	44.6 (6.07)	7.6 × 10^−7^
Duration of employment (years)	7.3 (7.44)	10.2 (8.66)	3.4 (1.30)	1.2 × 10^−22^
BMI (kg/m^2^)	25.3 (2.50)	25.4 (2.58)	25.3 (2.43)	0.944
QTc (msec)	401 (25.6)	398 (25.7)	405 (25.1)	0.003
QTi	97 (5.5)	96 (5.5)	99 (5.5)	0.0002
SBP (mmHg)	124 (12.3)	123 (11.9)	125 (12.7)	0.182
DBP (mmHg)	79 (7.8)	78 (7.9)	79 (7.5)	0.074
Categorical variables, N (%)				
Smoking habit				
Current smokers	115 (27.1)	65 (26.4)	50 (27.9)	0.729
Exsmoker/nonsmoker	310 (72.9)	181 (73.6)	129 (72.1)	
Shift schedule				
Fixed daytime shift (8–16)	297 (69.9)	192 (78.0)	105 (58.6)	1.8 × 10^−10^
H12	94 (22.1)	27 (11.0)	67 (37.4)	
H24	34 (8.0)	27 (11.0)	7 (4.0)	
Physical activity				
Regular	271 (63.8)	148 (60.2)	123 (68.7)	0.070
Irregular/never	154 (36.2)	98 (39.8)	56 (31.3)	
Alcohol intake				
Abstinent	223 (52.5)	122 (49.6)	101 (56.4)	0.287
Occasional	102 (24.0)	60 (24.4)	42 (23.5)	
Regular	100 (23.5)	64 (26.0)	36 (20.1)	
Stress dimensions, median score (IQR)				
Job demand	14 (12–18)	14 (12–17)	15 (12–18)	0.187
Job control	22 (19–24)	22 (19–24)	22 (19–24)	0.767
Job support	16 (15–18)	17 (15–19)	16 (15–18)	0.089

Note: BMI = Body Mass Index; QTc = Frequency-corrected QT; QTi = QT index; SBP = Systolic Blood Pressure; DBP = Diastolic Blood Pressure; H12 = 12 hour shifts; H24 = 24 hour shifts.

**Table 2:** Pearson’s correlation coefficients and Rho Spearman’s correlation coefficients (* = *p* < 0.05; ** = *p* < 0.01; s = Spearman’s coefficient).

Variables	QTc	QTi	Age	Duration of Employment	BMI	SBP	DBP	HR	Job Demand	Job Control	Job Support	Education	Location	Shift Work Schedule	Smoking	Alcohol	Physical Activity
QTc	1.000	0.959 **	0.115 *	−0.001	0.064	0.108 *	0.101 *	0.520 **	−0.035 ^s^	0.000 ^s^	0.074 ^s^	−0.116 * ^s^	0.147 ** ^s^	−0.047 ^s^	0.015 ^s^	−0.092 ^s^	−0.133 ** ^s^
QTi		1.000	0.120 *	−0.019	0.024	0.068	0.054	0.297 **	−0.054 ^s^	0.004 ^s^	0.061 ^s^	−0.101 * ^s^	0.183 ** ^s^	−0.075 ^s^	−0.006 ^s^	−0.094 ^s^	−0.079 ^s^
Age			1.000	0.326 **	0.174 **	0.189 **	0.290 **	0.024	0.057 ^s^	0.186 ** ^s^	−0.044 ^s^	−0.265 ** ^s^	0.248 ** ^s^	−0.120^* s^	−0.077 ^s^	0.047 ^s^	−0.061 ^s^
Duration of employment				1.000	0.022	0.018	0.078	0.123 *	0.075 ^s^	0.105 * ^s^	0.062 ^s^	−0.142 ** ^s^	−0.416 ** ^s^	−0.145 ** ^s^	−0.082 ^s^	0.089 ^s^	−0.015 ^s^
BMI					1.000	0.215 **	0.287 **	0.144 **	−0.018 ^s^	0.021 ^s^	0.017 ^s^	−0.128 ** ^s^	0.004 ^s^	−0.057 ^s^	0.073 ^s^	0.000 ^s^	−0.107 * ^s^
SBP						1.000	0.655 ^**^	0.153 **	0.019 ^s^	−0.008 ^s^	0.047 ^s^	0.001 ^s^	0.040 ^s^	0.087 ^s^	−0.041 ^s^	−0.009 ^s^	−0.088 ^s^
DBP							1.000	0.138 **	0.073 ^s^	0.038 ^s^	−0.040 ^s^	−0.130 ** ^s^	0.065 ^s^	−0.093 ^s^	−0.022 ^s^	0.078 ^s^	−0.125 ** ^s^
HR								1.000	0.023 ^s^	0.001 ^s^	0.068 ^s^	−0.133 ** ^s^	−0.061 ^s^	0.033 ^s^	0.072 ^s^	−0.023 ^s^	−0.211 ** ^s^
Job demand									1.000	−0.244 ** ^s^	−0.249 ** ^s^	−0.003 ^s^	0.064 ^s^	−0.030 ^s^	−0.014 ^s^	−0.010 ^s^	−0.049 ^s^
Job control										1.000	0.316 ** ^s^	−0.039 ^s^	−0.014 ^s^	−0.173 ** ^s^	−0.035 ^s^	0.051 ^s^	−0.097 * ^s^
Job support											1.000	0.054 ^s^	−0.083 ^s^	−0.061 ^s^	−0.070 ^s^	−0.060 ^s^	−0.092 ^s^
Education												1.000	−0.026 ^s^	−0.008 ^s^	−0.168 * ^s^	−0.048 ^s^	0.054 ^s^
Study group													1.000	0.209 ** ^s^	0.017 ^s^	−0.075 ^s^	0.088 ^s^
Shift work schedule														1.000	−0.007 ^s^	−0.020 ^s^	0.015 ^s^
Smoking															1.000	0.078 ^s^	−0.169 ** ^s^
Alcohol																1.000	0.039 ^s^
Physical activity																	1.000

Note: QTc = Frequency-corrected QT; QTi = QT index; BMI = Body Mass Index; SBP = Systolic Blood Pressure; DBP = Diastolic Blood Pressure; HR = Heart Rate.

**Table 3:** Multiple linear regression model predicting QTc in the whole study population and by operating location.

Covariates	Whole Study Population		Location 1		Location 2	
Variables	*β*	*p*-Value	*β*	*p*-Value	*β*	*p*-Value
Constant	387.428	0.000	380.108	0.000	424.796	0.000
Operating location	6.855	0.008	-	-	-	-
Physical activity	−6.947	0.007	−6.647	0.048	−8.143	0.047
SBP	0.159	0.119	0.204	0.144	0.117	0.442
Age	0.154	0.383	0.265	0.211	−0.101	0.759
Educational level	−4.205	0.113	−1.447	0.684	−8.216	0.043
Alcohol intake	−2.306	0.123	−2.796	0.152	−1.309	0.579
Fraction of the total variance explained by the model (R^2^)	0.049		0.027		0.025	

Note: SBP = Systolic Blood Pressure.

**Table 4:** Predicted QTc values and comparisons with results of the analysis of variance.

Karasek’s Categories	Low Job Support Mean Predicted QTc (95% CI)	High Job Support Mean Predicted QTc (95% CI)	*p*-Value *
Passive	400.42 (398.64–402.20)	400.85 (398.88–402.82)	0.742
Active	402.00 (400.06–403.94)	403.09 (400.67–405.51)	0.486
Low strain	402.23 (400.41–404.06)	400.64 (399.17–402.11)	0.187
High strain	400.61 (399.37–401.85)	401.52 (399.36–403.67)	0.434
*p*-value **	0.302	0.307	0.216 ***

Note: * ANOVA *p* values in the rows refer to the comparison of each Karasek’s category by job support (low vs. high). ** ANOVA *p* value in the columns refer to the comparison between the Karasek’s categories within the respective job support category. *** ANOVA *p* value refers to the comparison across the eight subgroups all together. QTc = Frequency-corrected QT.
